# Attraction of *Culex* mosquitoes to aldehydes from human emanations

**DOI:** 10.1038/s41598-017-18406-7

**Published:** 2017-12-21

**Authors:** Helena M. Leal, Justin K. Hwang, Kaiming Tan, Walter S. Leal

**Affiliations:** Department of Molecular and Cellular Biology, University of California-Davis, Davis, CA 95616 USA

## Abstract

Anecdotes related to preferential mosquito bites are very common, but to date there is no complete explanation as to why one out of two people systematically receives more mosquito bites than the other when both are equally accessible. Here we tested the hypothesis that two constituents of skin emanations, 6-methyl-5-heptan-2-one (6-MHO) and geranylacetone (GA), are natural repellents and may account for differential attraction in different ratios. We studied skin emanations from two human subjects, confirmed in behavioral assays that female southern house mosquitoes are significantly more attracted to subject A (attractant) than to subject N (non-attractant), and tested their 6-MHO/GA ratios in a dual-choice olfactometer. Although repelling at high doses, 6-MHO/GA mixtures were not active at the levels emitted by human skin. We found, however, differential attraction elicited by the aldehydes in the ratios produced by subjects A and N. When tested in a dose commensurate with the level released from human skin and in the ratio produced by subject A, the aldehyde mixture significantly attracted mosquitoes. By contrast, an aldehyde mixture at the same ratio released by subject N did not attract mosquitoes. We, therefore, hypothesized that aldehydes may play a role in the commonly observed differential attraction.

## Introduction

Female mosquitoes feed on humans and other vertebrates to acquire nutrients and unwittingly transmit disease-causing agents (e.g., viruses and pathogens) when their contaminated, needle-like mouthparts make contact with the victim’s blood stream^1,2^. Therefore, the public has a genuine interest in understanding how mosquitoes find their hosts. A Google search at the time of this writing, for example, produced almost 15 million results for the questions “why do mosquitoes find me?” Likewise, the question “why do mosquitoes prefer one person and not another?” generated more than 13 million results. Regardless of the public interest in the subject, scientists have yet to find out a definitive answer to these questions. It is well established, however, that host location is mediated by physical and chemical cues, particularly heat^3^, moisture^4^, visual cues^5^, and, more importantly, odorants (skin emanations)^6^. Evidence is growing in the literature suggesting that various factors contribute to differential attraction, including pregnancy^7,8^, malaria infection^9^, alcohol/beer consumption^10,11^, skin microbiota^12^, genetic makeup^13^, and even blood type^14^. Since the report almost a century ago identifying carbon dioxide (CO_2_) as a mosquito attractant^15^, CO_2_ has been used for trapping blood-seeking female mosquitoes. However, no evidence is available that suggests that CO_2_ mediates differential attraction, i.e., carbon dioxide emission levels do not explain the common observation that mosquitoes systematically prefer one person to another.

Investigators agree that skin emanations^16,17^ play a key role in the mosquito finding a host. These emanations are complex in nature and contain hundreds of compounds, but as far as mosquitoes are concerned a handful of compounds activate the mosquito’s olfactory system^18–22^. Of particular note, 6-methyl-5-hepten-2-one (sulcatone, hereafter 6-MHO), geranylacetone (hereafter, GA), octanal, nonanal, and decanal have been implicated in mosquito attraction and host shift^21^, repellency^23,24^, and even evolution of host preference^25^. Two of these compounds, namely 6-MHO and GA, have been hypothesized to be natural repellents^23,24^. Put this hypothesis simply, all skin emanations would, in principle, attract mosquitoes, but individuals producing 6-MHO and GA in appropriate ratios would produce a natural repellent that counters the attractants. Here, we report the findings of our studies aimed at testing this natural repellency hypothesis. We compared two human subjects, designated subjects A and N, to reflect the reported observation that subject A (for attractant) attracted more mosquitoes than subject N attracted. By analyzing their skin emanations and measuring the behavior of female southern house mosquitos in response to synthetic blends or their “intact” body odors, we confirmed that 6-MHO/GA mixtures indeed repel mosquitoes, but the amounts and ratios of 6-MHO/GA produced by the study subjects do not explain their differential attraction. Interestingly, however, we found that when tested at the level found in skin emanations, the aldehydes octanal, nonanal, and decanal elicited mosquito attraction when presented in the ratio produced by subject A, but not in the ratio produced by subject N.

## Results and Discussion

**Figure 1:**
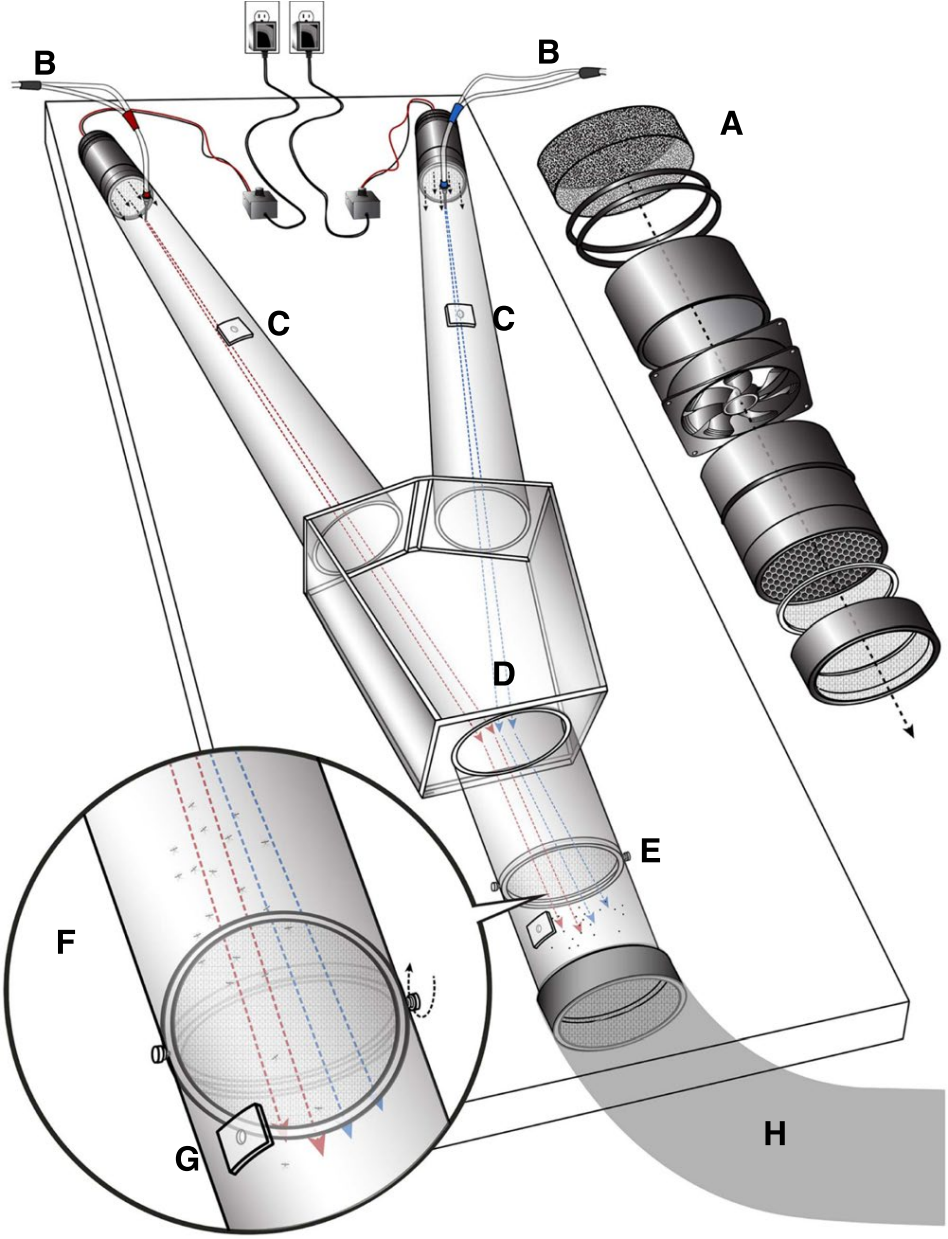
Diagrammatic representation of the newly constructed dual-choice olfactometer. (**A**) Exploded view of air filter, motor, and honeycomb system, (**B**) stimulus delivery system, (**C**) orifices for anemometer probe, (**D**) decision chamber, (**E**) rotating door of the release cage, (**F**) expanded view of the cage, (**G**) mosquito insertion hole, and (**H**) exhaustion system.

To test responses of the southern house mosquito, *Culex quinquefasciatus*, to skin emanations, we reasoned that the likelihood of success would increase if subjects were separated from the responding mosquitoes. It is customary to place a subject’s hand in the airflow path of a Y-olfactometer when testing responses of the yellow fever mosquito, *Aedes aegypti*, and typically these experiments lead to clear, consistent results^13^. Our preliminary experiments suggest that disrupting plume structures affects *Cx. quinquefasciatus* mosquito upwind flight responses. Another advantage of having subjects and mosquitoes in separate locations is that visual and other physical clues are eliminated. Therefore, we constructed a new dual-choice olfactometer to generate laminar flow even when live skin emanations were to be tested (Fig. 1). Improvements allow the plume to merge in the center of the downwind arm (Video 1). To eliminate the effect of CO_2_ and to activate mosquitoes, all experiments were performed with 15 mL CO_2_/min being delivered to each arm of the olfactometer. Blank tests showed that the arena is unbiased, given that there was no significant difference in the responses to the left and right sides of the olfactometer (mean ± sem, 46.5 ± 2.2 vs. 53.5 ± 2.2; *n* = 11; P = 0.1377; two-tailed, paired t-test). In these experiments, we released 10–15 mosquitoes per trial, with an average response of 87.9 ± 4.5%. In absolute numbers, 5.73 ± 0.51 vs. 6.34 ± 0.56 mosquitoes responded to left and right, respectively out of an average 12.6 ± 0.47 mosquitoes released per trial. Because the number of mosquitoes released per trial differed, it is more accurate to present the data in percentage responses, but the original raw data are reported (Supplemental Dataset 1).

**Figure 2:**
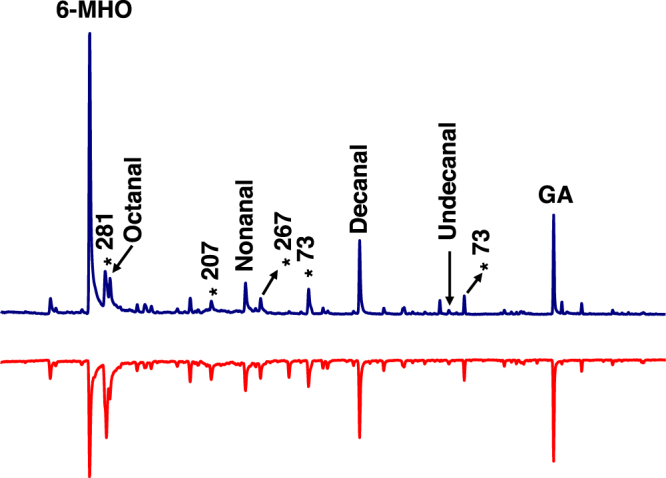
Gas chromatography-mass spectrometry traces. Skin emanations were collected from subject A (upper, blue trace) and subject N (lower, red trace) with SPME syringes placed on the right arm and covered with aluminum foil. Collection time: 30 min. Peaks derived from bleeding of the syringe fiber are denoted with asterisks and their base peaks in their mass spectra are indicated.

**Figure 3:**
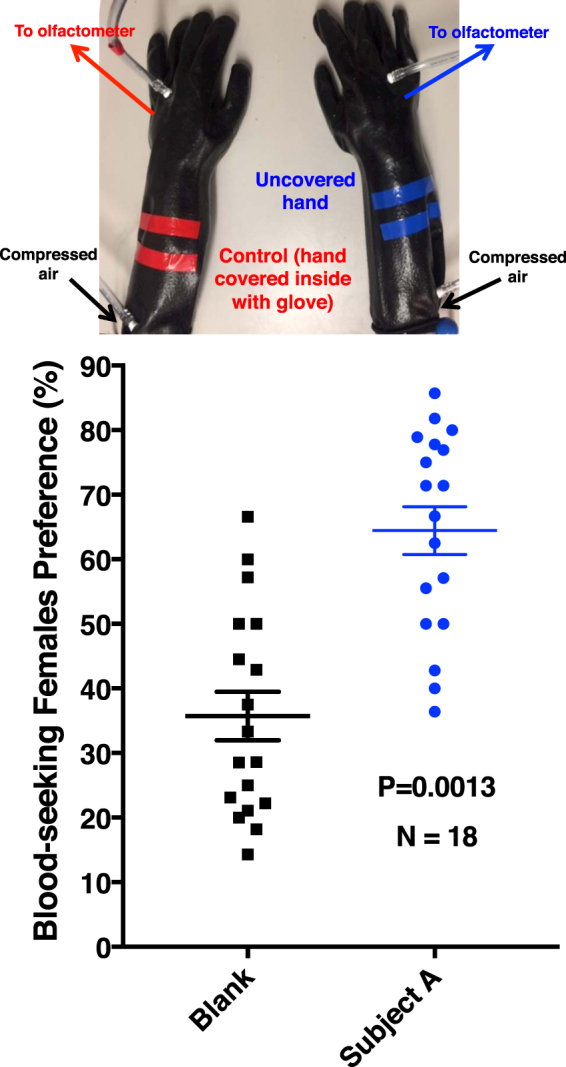
Behavioral responses from the southern house mosquito in a dual-choice olfactometer. The left hand/arm of the subject were covered (not shown) to serve as blank control, and the right hand/arm were uncovered to serve as a human source. After the gloves were tightly connected to the arms and sealed with Parafilm, two independently controlled lines of compressed air passed through the gloves and were connected to the decision arms of the olfactometer through the stimulus delivery pipettes.

**Figure 4:**
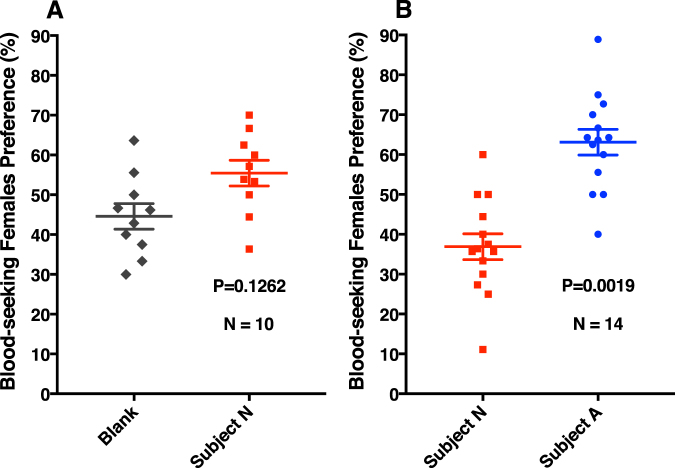
Behavioral responses from the southern house mosquito in a dual-choice olfactometer. (**A**) The right hand of subject N was used as stimulus and the left hand as control (blank). (**B**) Attractiveness of subjects A and N were directly compared.

**Figure 5:**
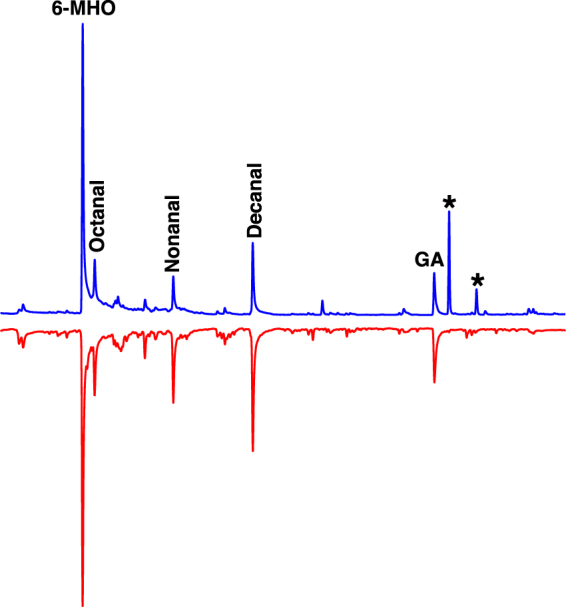
Gas chromatography traces from subjects A and N. Skin emanations from subject A (upper, blue trace) and subject N (lower, red trace) were collected from their left arms while their attractiveness was directly compared in behavioral studies (see above). Two peaks denoted with asterisks were contaminations from sunscreen applications to other parts of the body. Skin emanations were collected for 30 min.

**Figure 6:**
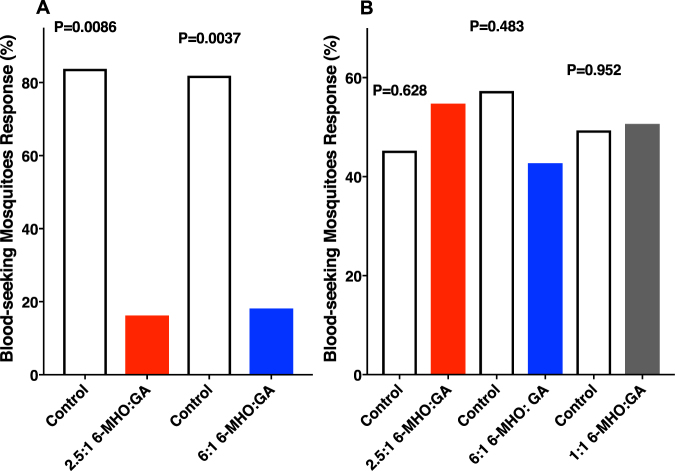
Repellency assays performed with human-derived repellents. Blends of 6-MHO/GA in the proportions found in subject N (2.5:1 6-MHO:GA) and subject A (6:1 6-MHO:GA) were tested in our surface landing and feeding assay at (**A**) 1% and (**B**) 0.1%. Because no repellency activity was observed at the lower dose, the optimal ratio^23^ (1:1 6-MHO:GA) was also tested.

**Figure 7:**
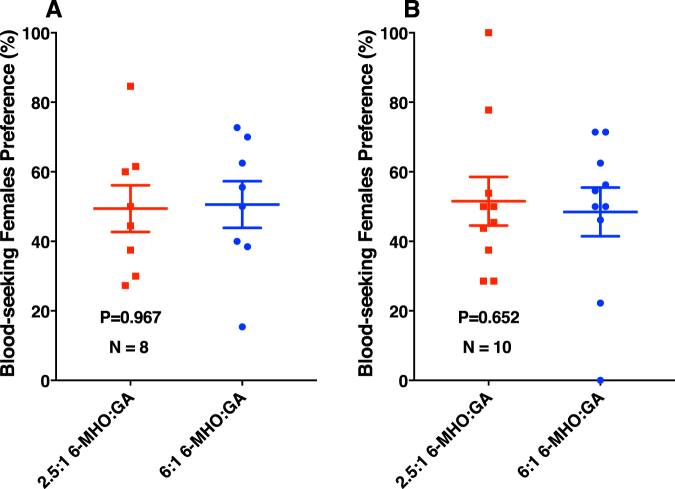
Behavioral responses of the southern house mosquito in a dual-choice olfactometer. Blends of 6-MHO/GA in the ratios produced by subjects A and N were directly compared at (**A**) 0.1% and (**B**) 0.01% to determine whether these natural repellents attract at lower doses.

Comparison of the human emanations by solid-phase microextraction (SPME) combined with gas chromatography-mass spectrometry showed that both subjects produced the five compounds (Fig. 2) previously reported to be associated with less-attractive individuals^24^. Specifically, GC-MS traces showed 6-MHO, octanal, nonanal, decanal, and GA produced in similar amounts, but different ratios. We then measured mosquito responses to these subjects compared to control or to each other. Here airborne volatiles (skin emanations) collected in a remote location were delivered in real time (“on-the-fly”) to the olfactometer. To keep the two sides of the olfactometer identical, independently controlled and adjusted airflow passed at 30 mL/min through each of the subject’s hands, with one hand/arm covered with a glove plus plastic film and the other uncovered and finally delivered (along with CO_2_) to the two choice arms of the arena. Of the 9.78 ± 0.7 mosquitoes released per trial, 89.2 ± 4.4% responded, and they were significantly (P = 0.0013, *n* = 18, two-tailed, paired t-test) more attracted to the side of the arena corresponding to the uncovered arm of subject A (Fig. 3). By contrast, there was no significant difference in the responses to the covered or uncovered arm of subject N (P = 0.1261, *n* = 10, two-tailed, paired t-test) (Fig. 4A). Here 89.5 ± 4.5% of the released 10.9 ± 0.64 mosquitoes per trial responded. Lastly, we compared mosquito responses when provided with airborne volatiles from subject N vs. subject A. Out of 11.64 ± 0.48 mosquitoes released per trial, 94.1 ± 2.7% responded, and they significantly (P = 0.0019, *n* = 14, two-tailed, paired t-test) preferred the arm connected to subject A’s skin emanations (Fig. 4B). Of note, the side of the arena as mentioned here is not a static physical side, because the stimulus was rotated between left and right for each trial, i.e., the stimulus Pasteur pipettes were rotated from trial to trial. To avoid unforeseen differences, when two subjects were tested at the same time, both airborne volatile collections were made with right hand gloves. Simultaneously, we collected airborne volatiles from the subjects in real time by placing SPME syringes on their left arms. Because the skin emanations were already identified and considering that gas chromatography is preferred for quantification over GC-MS, we analyzed these samples by GC (Fig. 5). We detected again a clear difference in the ratio of the target compounds in the profiles of the two subjects. To determine these ratios more accurately, we repeated these SPME collections. These analyses showed that the 6-MHO/GA ratios in subject N and subject A were 2.5:1 and 6:1, respectively. Although neither of these two subjects had the suggested optimal ratio for natural repellency (1:1)^24^, we surmised that a further detour from the optimal ratio as in subject A’s skin emanations could lead to “less repellency” and, consequently, more mosquito attraction. Then, we first compared repellency elicited by these two blends at 1% using our previously reported surface landing and feeding assay^26^. Both blends showed significant repellence (Fig. 6A), but no apparent difference occurred between them. As previously reported, differences in repellency by different ratios may not be noticeable when tested at higher doses.^26^ We then tested these two blends and the optimal ratio (1:1) at a lower dose, 0.1%. None of the blends had a significant difference compared to their respective controls (Fig. 6A). We then concluded that compositions of 6-MHO/GA do indeed repel mosquitoes, but the higher doses required (as in the case of most repellents) does not explain the differential attraction observed for subjects A and N. It is common knowledge in chemical ecology that certain compounds may repel at higher doses, but attract at lower doses. We then tested in our dual-choice olfactometer whether these blends of 6-MHO/GA at lower doses would attract mosquitoes. First, we tested the lowest dose that did not show repellence, i.e., 0.1%, which is sometimes referred to as 10^−3^ or 1,000 ppm. There was no significant difference (P = 0.967, *n* = 8, two-tailed, paired t-test) in the mosquito responses to the two blends (Fig. 7A), with 81.2 ± 5.4% of the 12.75 ± 0.67 mosquitoes released per trial responding. We then tried a lower concentration: 0.01% ( = 10^−4^ or 100 ppm), which is similar to the concentration estimated to be eluted from humans^25^. Again, there was no significant difference (P = 0.652, *n* = 10, two-tailed, paired t-test; 82.1 ± 7.9% response; 11.3 ± 0.89 mosquitoes released per trial) in the mosquito responses to these two doses (Fig. 7B), thus suggesting that these blends do not account for the differential attraction observed in our study.

**Figure 8:**
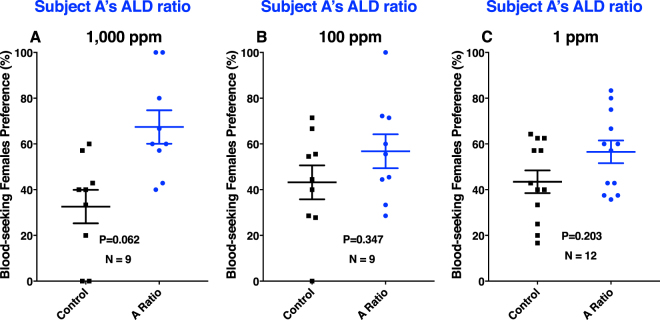
Behavioral responses of *Cx. quinquefasciatus* to mixtures of aldehydes. Mixtures of aldehydes in the ratio found in skin emanations from subject A (A Ratio), i.e., nonanal/octanal/decanal (1/1.25/2.5) were tested at (**A**) 1,000 ppm, (**B**) 100 ppm, and (**C**) 1 ppm. Although more mosquitoes were attracted to the treatment side of the arena in all doses, responses were not significantly different (P > 0.05).

**Figure 9:**
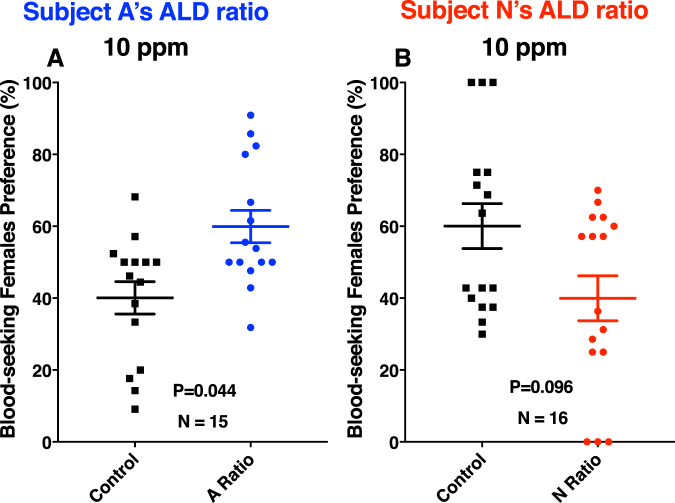
Behavioral responses of *Cx. quinquefasciatus* to mixtures of aldehydes at 10 ppm. Mixtures of aldehydes at (**A**) subject A’s ratio (A Ratio, i.e., nonanal/octanal/decanal; 1/1.25/2.5) and (**B**) subject N’s ratio (N Ratio, i.e., octanal/nonanal/decanal; 1/1.5/2.6) were tested in a dual-choice olfactometer. There was a significant difference in the responses to subject A’s ratio compared to control (**A**). By contrast, more mosquitoes were attracted to the control side than to the aldehyde blend at subject N’s ratio (**B**), but the difference was not significantly different.

Previously, we have demonstrated based on field experiments that nonanal is a mosquito attractant^21^. We then asked whether the mixtures of aldehydes in different ratios would show a differential attraction. With authentic standards and calibration curves, we determined that the ratios of the three aldehydes in the two subjects differed considerably. Subject A’s aldehyde ratio (A Ratio) was nonanal/octanal/decanal, 1:1.25:2.5, whereas subject N’s aldehyde ratio (N Ratio) was octanal/nonanal/decanal, 1:1.5:2.6. We tested mosquito responses at 1,000 ppm ( = 0.1% or 10^−3^) and found no significant difference between treatment and control (P = 0.062, *n* = 9, two-tailed, paired t-test) (Fig. 8A), although it did not escape our attention that there was a tendency towards the side of the arena with the aldehyde mixture. Interestingly and in marked contrast with other experiments, only 55.3 ± 5.9% of the released mosquitoes (9.77 ± 0.66 mosquitoes per trial) responded. Likewise, mosquitoes did not show a significant preference when tested at 100 ppm (P = 0.347, *n* = 9, two-tailed, paired t-test) or 1 ppm (P = 0.203, *n* = 12, two-tailed, paired t-test) (Fig. 8B,C). However, when tested at 10 ppm, mosquitoes significantly preferred the treated side of the arena (P = 0.044, *n* = 15, two-tailed, paired t-test) (Fig. 9A). Out of 14 ± 1.2 mosquitoes released per trial, 79.5 ± 7.6% of the mosquitoes responded. Of note, the total amounts of aldehydes are consistent with their amounts in skin emanation. In a 30-min airborne volatile collection from subject A’s arm, we detected ca. 80 ng of nonanal, whereas in these experiments we loaded a total of 100 ng (10 μL of a 10 ppm solution) of the blend. Mosquito responses to subject N’s aldehyde ratio at the same dose were noticeably different. First and foremost, there was no significant difference (P = 0.096, *n* = 16, two-tailed, paired t-test) (Fig. 9B), although there was a slight preference for the control side of the arena. Secondly, of the 13.6 ± 0.67 mosquitoes released per trial, 62.7 ± 6.8% responded. Given the apparent repellency (higher preference for the control side of the arena), although not statistically substantiated, we tested this blend at a lower dose (1 ppm). Again, there was no significant difference between treatment and control (P = 0.496, *n* = 10, two-tailed, paired t-test). Lastly, we tested 6-MHO alone at two different concentrations in our olfactometer. No significant differences occurred between treatments and controls (10 ppm, 43.8 ± 9.8 vs. 56.2 ± 9.8 control; P = 0.48, 100 ppm, 50.4 ± 7.9 vs. 49.6 ± 7.9 control; P = 0.914, *n* = 7 for both tests, two-tailed, paired t-test) We, therefore, hypothesize that the differential ratio of aldehydes is what might contribute at least in part to the differential attraction to *Culex* mosquitoes. Other factors, including but not limited to olfactory learning^27^, may play additional roles in differential mosquito attraction. We are cognizant of the difficulties inherent in large-scale human subject studies, but the light shed on this intriguing question by our study would justify the effort.

## Materials and Methods

### Mosquitoes

*Cx. quinquefasciatus* mosquitoes used in this study originated from Dr. Anthon Cornel’s stock laboratory colony, which in turn started from adult mosquitoes collected in Merced, CA, in the 1950s. The stock colony has been maintained in the Kearney Agricultural Center (Parlier, CA), whereas the Davis colony has been kept in our laboratory for more than six years. In Davis, mosquitoes were maintained at 27 ± 1 °C, 75% ± 5 relative humidity, and under a photoperiod of 12:12 h (light:dark). For behavioral assays, non-blood-fed female mosquitoes (6–7 days old) were separated into aluminum collapsible field cages (30.5 × 30.5 × 30.5 cm) with green polyester covers (Bioquip, Rancho Cordova, CA) the day before the experiments and provided with sugar and water. For repellency assays, 50–150 mosquitoes were transferred to the surface landing and feeding arena^26^ 30 min prior to starting behavioral observations. For bioassays conducted in the dual-choice olfactometer (see below), mosquitoes were transferred to the arena and allowed to acclimate for 1 min. Here, we tested a total of 1601 female mosquitoes (total n = 132), which were released in groups of 6 to 22 females, with a mean of 12 females per trial. The number of trials and percentage response per experiment are provided with figures.

### Human subjects

Two human subjects participated in this study, namely, a 17- year-old Latina and a 63-year-old Latino who are hereafter referred to as “Subject A” (for attractant) and “Subject N” (for non-attractant), given their own observation that when both are equally accessible, subject A receives more mosquito bites than subject N. They also noticed that subject N used to attract more mosquitoes, but of late he is bitten only when in areas of high mosquito densities and another host is not available. All experiments were performed in accordance with relevant guidelines and regulations, reviewed, and approved by the UC Davis Institutional Review Board (IRB). Informed consents and an assent were obtained from the subjects.

### Chemicals and chemical analysis

6-methyl-5-hepten-2-one (IUPAC name: 6-methylhept-5-en-2-one; also known as sulcatone), octanal, nonanal, decanal, undecanal, and titanium chloride were purchased from Sigma Aldrich (Milwaukee, WI) and geranyl acetone (IUPAC name: (5*E*)-6,10-dimethylundeca-5,9-dien-2-one) was acquired from Fluka via Sigma-Aldrich. Stock solutions (10% m/v) were prepared in hexane from which decadic dilutions were made for behavioral assays and calibration of gas chromatographic responses (see below). Gas chromatography/mass spectrometry (GC-MS) was performed on a 5973 Network Mass Selective Detector linked to a 6890 GC Series Plus + (Agilent Technologies, Palo Alto, CA), which was equipped with an HP-5MS capillary column (30 m × 0.25 mm; 0.25 µm film; Agilent Technologies). The oven temperature was programmed to start at 50 °C for 1 min and subsequently increased to 250 °C at a rate of 10 °C/min, with a final hold of 5 min. Another program started at 50 °C for 1 min, but first increased at 5 °C/min to 100 °C before a ramp of 10 °C/min to 250 °C (holding time 5 min). After each run, the oven temperature was set at 290 °C for 5 min. The injector was equipped with a 0.75 mm ID Solid phase microextraction (SPME) injection sleeve (Supelco, Bellefonte, PA) and operated at 250 °C in pulsed splitless mode. MS transfer line was set at 280 °C and MS quad and MS sources were set at 150 °C and 230 °C, respectively. Quantification was done on a gas chromatograph 6890 Series GC (Agilent Technologies) equipped with an HP-5MS, i.e., the same type of column used in GC-MS. The oven was operated in different temperature programs: one starting at 70 °C for 1 min and increased at a rate of 10 °C/min to 290 °C; another starting at 50 °C for 1 min and increased at a rate of 10 °C/min to 250 °C. The final temperature of the short versions of these programs was set at 200 °C. The injector with an SPME injection sleeve (see above) and the flame ionization detector (FID) were operated at 250 °C. FID responses were calibrated with authentic standards to generate the following curves: y (amount of 6-methyl-5-heptan-2-one in ng) = 0.057X-1.525; X = peak area; R^2^ = 0.957; y (amount of decanal in ng) = 0.161X-1.898; X = peak area; R^2^ = 0.964; y (amount of nonanal in ng) = 0.088X + 0.266; R^2^ = 0.918; y (amount of geranylcetone in ng) = 0.056X-0.269; X = peak area; R^2^ = 0.947. Skin emanations were collected with SPME 50/30 mm DVB/Carboxen/PDMS Stable Flex (Supelco, catalogue number 57328-U) using a previously reported protocol^21^. Subjects avoided using cosmetics and/or sunscreen starting the day before the experiments. Prior to volatile collections, they washed their arms extensively with distilled water for at least 5 min. To estimate the amounts of aldehyde released from the subjects, we collected airborne volatiles from a “hand in glove” using N_2_ and with the outlet of the flow passing through a small SuperQ (Alltech, Deerfield, IL) column. After 30 min, the column was washed with hexane, and the extract was concentrated and analyzed by GC and GC-MS.

### Repellence assay

Repellence was measured in the previously described surface landing and feeding assay^26^. Two hundred microliters of the test samples or the control, hexane only, were loaded in filter paper rings and tested within 3 min. Once assay started, the experimenters left the room and returned after 5 min. End-point responses were measured by counting the number of females feeding on cotton rolls loaded with blood. Females that crossed the “repellent curtain,” went inside the arena, but were not feeding at the end of assay were not counted. Fresh cotton rolls and filter paper rings were prepared for each run.

### Dual-choice olfactometer

The design of our new olfactometer (Fig. 1) was inspired by a recent prototype^28^, which in turn considered features of previous^29^ models, particularly an original setup: “the grandfather” of all modern olfactometers^30,31^. A couple of improvements over our previous model^32^ are the use of two independently controlled motors to push air through the decision arms rather than having a single fan behind a release chamber and the angle of the arm combined with the shape of the decision box. The new model differs markedly from our previous setup^32^ in that air was pushed into the olfactometer through two independently controlled motors at the downwind end, as opposed to a single motor that pulled air in the previous version^32^. It differs slightly from the model that inspired us^33^ and our own earlier model^32^ in that the two choice arms do not connect to the decision chamber (Fig. 1D) at the right angle. To maintain the filamentous structure of the plumes while merging inside of the decision chamber, the decision arms were set at an angle of ≈35° of each other, as suggested by Dr. J. Riffell (University of Washington). The decision chamber (rectangular, downwind face: 14 cm × 12 cm height; length of the chamber, 23 cm; upwind faces: 13.5 cm × 12 cm height) was made of Plexiglas and permanently connected to two Plexiglas cylinders (81 cm long; 9.2 cm internal diameter). Each of these two choice arms had an opening (6 mm internal diameter) at 50 cm from the decision chamber to allow introduction of a probe to measure air velocity (Anemomaster, model 6006–2 G, Kanomax, Osaka, Japan). The arm at the downwind end of the decision chamber housed a previously described^32^ releasing cage (Fig. 1F) with a wire mesh rotating door (Fig. 1E), which was centered at 68 cm from the entrance of the decision chamber and connected to an exhaustion system (Fig. 1F). Mosquitoes were introduced into the arena through an opening (1 cm, ID) in the releasing cage. All openings were sealed with Parafilm when not in use. Stimuli and controls were delivered through Pasteur pipettes, which were set at 3 cm from the upwind end of each arm. Pipette tips (1 mL) were cut at 12 mm from the top and inserted into each arm to house the Pasteur pipettes and allow the pipette tips to be placed exactly at the center of the flow. That way the filamentous plumes were not disturbed by stimulus delivery (Video 1). The two independent airflow systems comprised a motor (Flight LT brushless DC motors, Comair Rotron, Shanghai, China), which was attached to recycled pipes (14.5 cm long; 7.8 cm ID) from BioQuip’s gravid mosquito trap. The other end of the pipe housed a honeycomb (1/4 ACG NP EXP 2.000 × 40”X40”; HoneyCommCore LLC, Mills River, NC, USA), which was handcrafted to fit tightly into the pipe. A 4-cm long Acetyl-Butyl-Styrene (ABS) pipe (4 cm long; 7.8 cm ID) was attached behind the motor and covered with double layers of carbon filter fabric strips (P/N 87365K24; McMaster-Carr, Santa Fe Springs, CA, USA), which in turn were secured with oil-resistant Buna-N O-rings (McMaster-Carr; P/N 9452K98). A 2-cm ABS sleeve was attached in from of the honeycomb to house a circular wire mesh to further improve the quality of the plume and prevent further upwind movement by mosquitoes. The complete motor systems were housed into Plexiglas pipes (18 cm long; 9.2 cm ID) and secured with screws. They were attached to the arm of the olfactometer and covered with 6-cm long (10.1 cm ID) Plexiglas pipes so as to make it easy to disassemble for cleaning. The motors were powered by power supplies (12 VDC, 1 A) and controlled independently by pulse with modulation motor speed controllers (MXA033, Maxx Tronic/Carl’s Electronics, Oakland, CA, USA) for fine adjustments. Stimuli were delivered (Fig. 1B) with airflows of carbon dioxide (15 mL/min) and combined with a flow of compressed air when live subjects were tested. The carbon dioxide and the air lines merged into tubes, which were capped with pipette tips cut at 15 mm from the top to serve as sleeves for Pasteur pipettes used to load stimuli. When live subjects were tested, clean Pasteur pipettes were used. For other experiments, strips of filter paper (7 cm × 0.3 cm) were loaded with 10 μL of defibrinated sheep blood (University of California-Davis, Biological Media Services, Cat # 4024) plus 10 μL of hexane (control) or 10 μL of a test solution. For live subjects, a remote delivery system was constructed. This is reminiscent of the skin-odor tube previously reported^34^ for wind tunnel experiments, except that in our dual-choice setup a perfect flow balance is necessary to avoid bias in mosquito responses. Two large chemical resistant, vinyl coated gloves (Magid Glove & Safety Mfg. Co LLC, China; Catalogue number T1088RT) were modified to have two hose inlet glass adapters, one 9 cm away from the arm end of the glove (inlet) and another just above the compartment for fingers (outlet). They were tightly connected to the gloves with Teflon tape. The hose inlets were connected to VWR clear, flexible tubing (# 60985–528). Air (ZERO Grade, Praxair, Danbury, CT) was delivered from a cylinder equipped with a two-stage regulator (P/N 5183–4645; Agilent Technologies). The outlet was split into two separate regulators with 60 psi Gauge (P/N 23831-U; Supelco, Bellefonte, PA), and each independent line was fed into one of the gloves. When in use, the opening end of each glove was attached to the subject’s arm, secured with 30-cm bungee cords (P/N 06344; Keeper, Foothill Ranch, CA, USA), and tightly sealed with Parafilm. Each outlet was connected to a 50-mL Dudley bubbling tube (P/N 40356, Fisherbrand) loaded with 10 mL of double distilled water. The outlet of the tubes was connected to VWR clear, flexible tubing, passed through small holes in the wall and finally connected to the olfactometer (Fig. 1B) located in the next-door room. Airflow, measured at the outlet of the Dudley tubes, was adjusted to 30 mL/min just before experiments started. During behavioral assays, the olfactometer room was kept in the dark and maintained at relative humidity of 65–75%, and at a temperature ranging from 27 to 30 °C. Experiments were conducted from 16:00 to 18:00, ie, at the beginning of the scotophase. The subjects were placed in a room with air conditioning and regular office light. Mosquito responses were observed in real time with a Night Vision Headlamp (3 pcs XML T6; Boruit; purchased through amazon.com). Given that they respond immediately after the door is opened, at least two observers were required for each experiment. A positive response was recorded when mosquitoes passed the decision chamber and crossed one of the arms of the olfactometer. Most mosquitoes that made a decision continued to fly upwind until they reached the net protecting the motor. It was not uncommon to observe mosquitoes returning to the decision chamber and making a second choice to the same or different side of the arena. Therefore, the recorded percentage of responses might be somewhat biased by dual responses from the same individual. The olfactometer was cleaned with SNOOP liquid leak detector (Sigma-Aldrich) diluted with distilled water.

## Electronic supplementary material

Supplementary Information

Supplementary Video 1
