# Supplementary material for: Attraction of Culex mosquitoes to aldehydes from human emanations
Source: Sci Rep. 2017 Dec 21;7:17965. doi: 10.1038/s41598-017-18406-7 (PMC5740115; doi:10.1038/s41598-017-18406-7)
Supplement: Supplementary file 1 — Supplementary Information [file 41598_2017_18406_MOESM1_ESM.pdf]

Attraction of *Culex* mosquitoes to aldehydes from human emanations

Helena M. Leal, Justin K. Hwang, Kaiming Tan & Walter S. Leal*

Department of Molecular and Cellular Biology, University of California-Davis, Davis CA 95616 USA

*Corresponding author:

Walter S. Leal

wsleal@ucdavis.edu

Video 1. Illustration of the laminar flow generated in the olfactometer. Plumes were traced by delivering titanium chloride through the stimulus Pasteur pipettes. For clarity and to minimize reflection, red cardboard papers were place behind the arms and decision chamber.
